# Numerical investigation of effects of tongue articulation and velopharyngeal closure on the production of sibilant [s]

**DOI:** 10.1038/s41598-022-18784-7

**Published:** 2022-09-13

**Authors:** HsuehJui Lu, Tsukasa Yoshinaga, ChungGang Li, Kazunori Nozaki, Akiyoshi Iida, Makoto Tsubokura

**Affiliations:** 1 Computational Fluid Dynamics Laboratory, Department of Computational Science, Graduate School of System Informatics, Kobe University, 1-1 Rokkodai, Nada-ku, Kobe, 657-8501 Japan; 2 Complex Phenomena Unified Simulation Research Team, RIKEN, Advanced Institute for Computational Science, Kobe, 650-0047 Japan; 3 Toyohashi University of Technology, 1-1 Hibarigaoka, Tempaku-cho, Toyohashi, Aichi 441-8580 Japan; 4 Osaka University Dental Hospital, 1-1 Yamadaoka, Suita, Osaka 565-0871 Japan

**Keywords:** Computational models, Biomedical engineering

## Abstract

A numerical simulation of sibilant /s/ production with the realistically moving vocal tract was conducted to investigate the flow and acoustic characteristics during the articulation process of velopharyngeal closure and tongue movement. The articulation process was simulated from the end of /u/ to the middle of /s/ in the Japanese word /usui/, including the tongue elevation and the velopharyngeal valve closure. The time-dependent vocal tract geometry was reconstructed from the computed tomography scan. The moving immersed boundary method with the hierarchical structure grid was adopted to approach the complex geometry of the human speech organs. The acoustic characteristics during the co-articulation process were observed and consistent with the acoustic measurement for the subject of the scan. The further simulations with the different closing speeds of the velopharyngeal closure showed that the far-field sound during the co-articulation process was amplified with the slower closing case, and the velum closure speed was inverse proportional to the sound amplitude with the slope value of − 35.3 dB s/m. This indicates possible phonation of indistinguishable aeroacoustics sound between /u/ and /s/ with slower velopharyngeal closure.

## Introduction

Speech disorders impact directly a person’s social activities and career. In Japan, the number of children who have articulation disorder is estimated approximately 556,000; the number of adults who have articulation disorder is estimated approximately 293,000; the number of people who have stuttering is estimated approximately 710,000 in 2016^1^. Although the articulation disorder can be caused by a variety of reasons, the current study put the emphasis on the morphological, and neurological articulation process and investigate the effect of tongue articulation and velopharyngeal closure on the speech production of sibilant /s/ with the numerical simulation.

The sibilant sound is one of the most difficult articulations in the consonant production, and it is known to acquire in the latest leaning process. Therefore, it is often reported in articulation disorders due to the insufficient tongue formation^2^. The sibilant /s/ is generated by the airflow passing through a constriction formed between the alveolar ridge and the tongue tip. When the airflow is forced to go through the narrow constriction (sibilant groove) of the tongue tip, the turbulent jet flow is generated, and the velocity fluctuation downstream from the constriction is considered as the source of the sound^3,4^. The sibilant fricatives are characterized as broadband noise in the frequency range above the characteristic peaks, approximately 4–7 kHz for /s/^5^.

According to Kummer^6^, the velopharyngeal dysfunction is a speech disorder that the velopharyngeal valve does not close consistently and/or completely during the production of oral consonants. The causes for the velopharyngeal dysfunction are usually reported as abnormal anatomy due to cleft palate which leads to the velopharyngeal insufficiency, abnormal neurophysiology which leads to the velopharyngeal incompetence, and functional articulation errors as the velopharyngeal mislearning. The velopharyngeal valve, which consists of the velum (soft palate) as well as the lateral and posterior pharyngeal walls, controls the amount of airflow going to the nasal cavity. During the normal nasal breathing or the nasal consonant productions, the soft palate rests, and the velopharyngeal valve opens the flow channel. Then, the airflow goes through the nasal cavity, which results in an acoustic coupling between the oral and nasal cavities during the nasal consonant productions. On the contrary, during the production of the oral consonants, such as fricatives and stops (e.g., /s/ and /p/), the complete closure of the velopharyngeal valve is required to increase the oral air pressure. Hence, the velum moves superior and posterior directions to close the velopharyngeal valve against the airflow going to the nasal cavity.

If the flow escapes through the nasal cavity with the velopharyngeal insufficiency during the sibilant sound production, the nasal sound emission occurs due to the jet flow generation at the valve. In this situation, the speech sound is distorted by an acoustic resonance in the nasal cavity. The effects of the velopharyngeal opening and the mechanism of the nasal emission have been investigated by the measurements^7,8^ and numerical simulations^9–12^. Oren et al.^13^ provided a brief review of the types of nasal emission and the potential causes, and they reported that when there is a characteristic velopharyngeal insufficiency, the audibility of nasal emission can give clues for the relative size of the valve opening. Kummer et al.^14,15^ compared the velopharyngeal gap size in patients with the different level of the perceived characteristics of speech and indicated that the degree of the velopharyngeal insufficiency can be predicted to some extent based on the perceptual assessment. Bunton et al.^7^ examined the relationship among perceptual ratings of the hypernasality, and noted the differences in nasalance, size of the nasal valve opening and perceptual ratings of hypernasality among the three English vowels, /i/, /u/, and /ɑ/. The results indicated that the listeners could detect the hypernasality for the high and low vowels with nasal port areas from 0.01 to 0.15 cm^2^, respectively.

Sundström et al.^9–12^ conducted a series of studies by utilizing the numerical flow simulations to investigate the effect of the velopharyngeal insufficiency. To determine the flow behaviour and the sound generating mechanism in the vocal tract, they conducted the simulations of /s/ with the different sizes of the velopharyngeal valve^9,10^. They confirmed that the turbulence in the nasal cavity indeed affected the speech sound and explained that the additional sound was generated by interaction of the turbulence with the solid nasal wall. In addition, to clarify the relationship between the flow characteristics in the nasal cavity and the size of the velopharyngeal opening, they conducted another numerical simulation by changing the size of the velopharyngeal opening^14,15^ and found that the flow velocity and the turbulence intensity were inversely proportional to the size of the opening if the flow resistance across the velopharyngeal valve was smaller than that across the oral constriction. The series of results indicated that the ratio of channel areas at the velopharyngeal valve and the oral constriction can be considered as a factor to determine the airflow configuration during the production of sibilant /s/ sound. However, almost all studies of the flow simulation used a static vocal tract geometry which only provides the stationary result instead of the time-varying sound production in the actual human speech process.

Therefore, in the current study, the numerical simulation of sibilant /s/ production with the realistically moving vocal tract was conducted to investigate the effect of velopharyngeal closure and tongue movement during the articulation process. The speech production process after the vowel /u/ to sibilant /s/ was simulated in the part of the Japanese word /usui/, which is an example of VCV sequence that induces natural movement of the tongue and velum closure. The flow and acoustic field during the phonation process were clearly revealed. Additionally, previous experimental observations indicated that the moving speed and timing of the speech organ is also an important factor in the sound production during the phonation process^16–18^. Hence, simulations with faster and slower closing velopharyngeal valve were conducted to investigate the effect of the velum closure speed during the sibilant sound. For the first step, the velopharyngeal valve motion was accelerated and decelerated from the original speed within the range of normal articulation speed. By analyzing the spectrogram of the predicted sound, the current study clarifies the effects of velopharyngeal closure speed during the articulation process.

## Methods

### Vocal tract geometry

**Figure 1:**
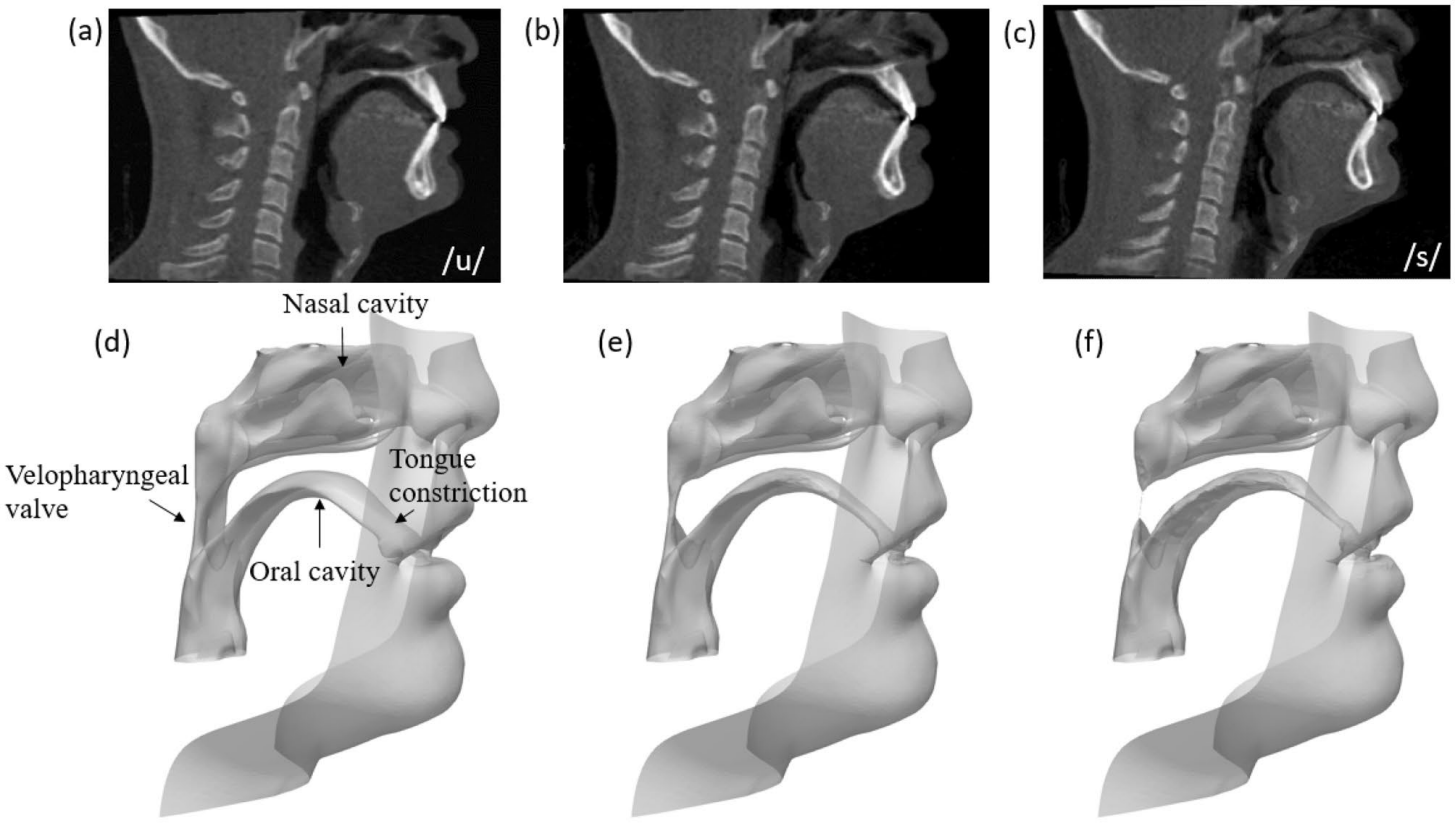
CT images and vocal tract geometry of (**a**) the end of vowel /u/ to (**c**) the sibilant sound/s/in the phonation process of /usui/.

To simulate the articulatory process, the geometry of a vocal tract was extracted from four-dimensional computed tomography (4D-CT) images of a subject pronouncing a Japanese word /usui/ (*“thin”* in English) which using VCV sequence including natural movement of the tongue and velum closure. The subject was a 42-year-old Japanese male who self-reported no speech disorders with a normal dentition of angle class I. The CT scan was conducted by a 320-row Area Detector CT (20 fps; 320 slices of 512 × 512 pixels). The subject pronounced the Japanese word /usui misosiru/ while the subject was sitting down. The vocal tracts were collected from the scans of the articulatory process and the geometries were extracted from the CT images based on brightness values using the open source software “elasti”^19^ using the insight segmentation and registration toolkit (ITK), as shown in Fig. 1, including the oral cavity, nasal cavity, tongue constriction and velopharyngeal valve. The geometries used in the current study were the duration from the end of vowel /u/ (Fig. 1a,d) to the sibilant sound /s/ (Fig. 1c,f), which includes the movement of tongue elevation and the closure of the velopharyngeal valve. During the simulation, the geometries at each time step were interpolated from these three geometries (Fig. 1d–f) to mimic the realistic motion of the vocal tract. All methods were performed in accordance with the relevant guidelines and regulations of the ethics committee. The informed consent was obtained from all subjects. The ethics committee of the graduate school of Osaka University certified this study (H26-E39).

**Figure 2:**
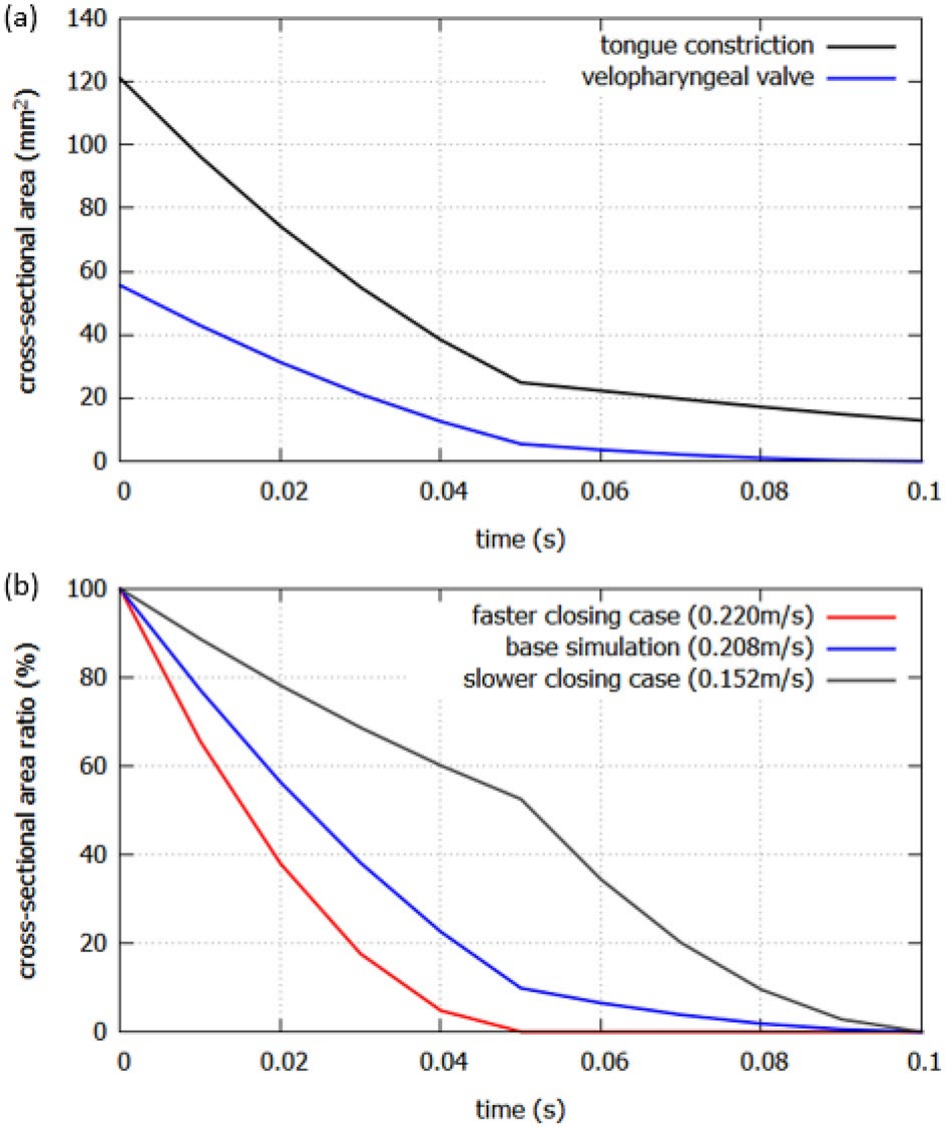
(**a**) The cross-sectional area of the tongue constriction and the velopharyngeal valve during the movement. (**b**) The cross-sectional area ratio of different closing speed.

The cross-sectional areas of the tongue constriction and the velopharyngeal valve during the movement are shown in Fig. 2a. The preliminary simulation with the stationary geometry was from *t* = − 0.005 s to *t* = 0 s to avoid the effects of unstable state on the results of the far-field sound. After *t* = 0.0 s, the cross-sectional areas of the tongue and velum constrictions are reduced from 121 mm^2^ and 55.6 mm^2^ to 12.9 mm^2^ and 0.01 mm^2^, respectively.

To investigate the effect of the velopharyngeal closure speed, the speed of the velum was changed by modifying the cross-sectional area of the velopharyngeal valve at the middle between /u/ and /s/ (*t* = 0.05 s). According to the previous study^11^, the ratio of area between the velopharyngeal valve and the oral constriction can be considered as a factor for the production of sibilant /s/. Furthermore, the most severe nasal emission was produced when the size of the velopharyngeal opening was equal to the size of the oral constriction. Therefore, the velum opening area was enlarged from 5.48 mm^2^ (original model) to 29.2 mm^2^ which is close to the area of oral constriction for the slower closing case, and reduced to 0.01 mm^2^ for the faster closing case. As shown in Fig. 2b, a faster velopharyngeal closure (red line) and a slower velopharyngeal closure (gray line) were simulated with the original closure speed (blue line). The initial and the end of the velopharyngeal valve size of all cases are set to be the same, however, the velopharyngeal channel was totally closed at *t* = 0.05 s in the faster case, while the channel was still opening 50% at *t* = 0.05 s in the slower case. The closure speeds of the velum are 0.220 m/s, 0.208 m/s and 0.152 m/s for three cases.

### Governing equation

The governing equations are the compressible Navier–Stokes equations:

1 $$ \frac{\partial U}{{\partial t}} + \frac{{\partial F_{1} }}{{\partial x_{1} }} + \frac{{\partial F_{2} }}{{\partial x_{2} }} + \frac{{\partial F_{3} }}{{\partial x_{3} }} = 0, $$

where *t* is the time, *x*_*i*_ indicates three directions in Cartesian coordinate system (*i* = 1, 2, 3), and the conservative vector *U* is

2 $$U={\left(\rho {\rho u}_{1} {\rho u}_{2} {\rho u}_{3} \rho e \right)}^{T},$$

where the flux vectors *F*_*i*_ are

3 $$ F_{i} = \left( {\begin{array}{*{20}c} {\rho u_{i} } \\ {\rho u_{i} u_{1} + P\delta_{i1} - \mu A_{i1} } \\ {\rho u_{i} u_{2} + P\delta_{i2} - \mu A_{i2} } \\ {\rho u_{i} u_{3} + P\delta_{i3} - \mu A_{i3} } \\ {\left( {\rho e + P} \right)u_{i} - \mu A_{ij} u_{j} - k\frac{\partial T}{{\partial x_{i} }}} \\ \end{array} } \right),\quad \forall \,\,\,i = 1,2,3, $$

*ρ* is the density, *u*_*i*_ is the velocity components (*i* = 1, 2, 3), and *δ*_*ij*_ indicates the Kronecker delta.

The total energy *e* is as

4 $$ e = \frac{P}{\rho (\gamma - 1)} + \frac{1}{2}\left( {u_{1}^{2} + u_{2}^{2} + u_{3}^{2} } \right), $$

and *µA*_*ij*_ is the stress term, while

5 $$ A_{ij} = \frac{{\partial u_{i} }}{{\partial x_{j} }} + \frac{{\partial u_{j} }}{{\partial x_{i} }} - \frac{2}{3}\left( {\nabla \cdot u} \right)\delta_{ij} . $$

The pressure *P* is given from the ideal gas equation:

6 $$ P = \rho RT. $$

The working fluid is air, and the computational condition is standard temperature and pressure^20^. The dynamic viscosity $\mu$ and thermal conductivity *k* follows Sutherland’s law:

7 $$ \mu \left( T \right) = \mu_{0} \left( {\frac{T}{{T_{0} }}} \right)^{\frac{3}{2}} \frac{{T_{0} + 110}}{T + 110}, $$

8 $$ k\left( T \right) = \frac{\mu \left( T \right)\gamma R}{{\left( {\gamma - 1} \right)\Pr }}, $$

where $\rho_{0}$ = 1.1842 kg/m^3^, $\mu_{0}$ = 1.85 10^−5^ N∙s/m^2^, *T*_0_ = 298.06 K, $\gamma$ = 1.4, *R* = 287 J/kg, and the Prandtl number (Pr) is 0.71.

The following numerical framework was adopted to solve the three-dimensional compressible flow governed by Eq. (1). We applied the second-order-accurate implicit lower–upper symmetric Gauss–Seidel scheme (LUSGS) for time integration. The Roe scheme^21^ with a preconditioning method and dual time stepping is adopted, and the discretized form of Eq. (1) with artificial time step Δτ is

9 $$  \Gamma \frac{{\bar{U}_{p}^{{k + 1}}  - \bar{U}_{p}^{k} }}{{\Delta \tau }} + \frac{{3\bar{U}^{{k + 1}}  - 4\bar{U}^{n}  + \bar{U}^{{n - 1}} }}{{2\Delta t}} + \frac{1}{{\Delta x_{1} }}\left( {\bar{F}_{{1\left( {i + \frac{1}{2},j,k} \right)}}^{{k + 1}}  - \bar{F}_{{1\left( {i - \frac{1}{2},j,k} \right)}}^{{k + 1}} } \right) + \frac{1}{{\Delta x_{2} }}\left( {\bar{F}_{{2\left( {i,j + \frac{1}{2},k} \right)}}^{{k + 1}}  - \bar{F}_{{2\left( {i,j - \frac{1}{2},k} \right)}}^{{k + 1}} } \right) + \frac{1}{{\Delta x_{3} }}\left( {\bar{F}_{{3\left( {i,j,k + 1/2} \right)}}^{{k + 1}}  - \bar{F}_{{3\left( {i,j,k - 1/2} \right)}}^{{k + 1}} } \right) = 0, $$

where *Г* is the preconditioning matrix proposed by Weiss and Smith^22^, *U*_*p*_ is the primitive form [*P*, *u*_*1*_, *u*_*2*_, *u*_*3*_, *T*], τ and *t* indicated artificial time and physical times, the superscripts *k* is the iteration numbers in artificial time step and *n* is the proceeding step of real time. The quantities associated with the artificial time term of the $(k + 1)$th iteration are transferred approximately to quantities of the $(n + 1)$th time step in real time when the term $\partial U_{p} /\partial \tau$ converges to zero. Then, Eq. (9) will be turned back to the original Navier–Stokes equation including the transient term.

Finally, Eq. (1) can be wrote as

10 $$ \left[\frac{I}{\Delta \tau } + \Gamma^{ - 1} M\frac{3}{2\Delta t} + \Gamma^{ - 1} (\delta_{{x_{1} }} A_{p}^{k} + \delta_{{x_{2} }} B_{p}^{k} + \delta_{{x_{3} }} C_{p}^{k} )\right]\Delta U_{p} = \Gamma^{ - 1} R^{k} , $$

where $M = \partial U/\partial U_{p}$, ${A}_{p}^{k}=\partial {F}_{1}^{k}/\partial {U}_{p}$, ${B}_{p}^{k}=\partial {F}_{2}^{k}/\partial {U}_{p}$, ${C}_{p}^{k}=\partial {F}_{3}^{k}/\partial {U}_{p}$ are the flux Jacobian, $R^{k} = - (3U^{k} - 4U^{n} + U^{n - 1} )/(2\Delta t) - (\delta_{{x_{1} }} \overline{F}_{1}^{k} + \delta_{{x_{2} }} \overline{F}_{2}^{k} + \delta_{{x_{3} }} \overline{F}_{3}^{k} )$, and $\delta_{{x_{i} }}$ is the central-difference operator.

The solution-limited time stepping (SLTS) method adaptively adjusting the CFL number and determine Δτ in the governing equation was proposed by Lian et al.^23^ to accelerate the convergence speed. When adopting LUSGS method, the estimation value Δ*Q*_*est*_ defined as.

11 $$ \Delta Q_{est} = - \Delta \tau M^{ - 1} [ - (3U^{k} - 4U^{n} + U^{n + 1} )/(2\Delta t) - (\delta_{{x_{1} }} \overline{F}_{1}^{k} + \delta_{{x_{2} }} \overline{F}_{2}^{k} )], $$

and Δ*Q*_*ref*_ is defined as.

12 $$ \Delta Q_{ref} = \left( {\begin{array}{*{20}l} {\alpha_{1} \times \max [0.5 \times \rho \left( {u_{1}^{2} + u_{2}^{2} } \right),\quad \Delta P_{sur} ,\quad P_{global} \times 10^{ - 9} ]} \hfill \\ {\alpha_{2} \times \max \left[ {\left( {u_{1}^{2} + u_{2}^{2} } \right),\quad \frac{{\Delta P_{sur} \times c}}{\gamma P}\quad V_{global} \times 10^{ - 9} } \right]} \hfill \\ {\alpha_{3} \times \max \left[ {\left( {u_{1}^{2} + u_{2}^{2} } \right),\quad \frac{{\Delta P_{sur} \times c}}{\gamma P}\quad V_{global} \times 10^{ - 9} } \right]} \hfill \\ {\alpha_{4} \times T} \hfill \\ \end{array} } \right) $$

where Δ*P*_*sur*_ indicates the maximum difference between the pressure at surrounding points and *P*_*global*_, *V*_*global*_ denotes the global value that ensures the reference values are always greater than 0, c is the speed of sound, and *γ* is the heat capacity ratio. Equation (12) provide a criterion for determining the stability of the calculation. According to Lian^23^, the factor [*α*_*1*_* α*_*2*_* α*_*3*_* α*_*4*_] is the coefficient of the maximum allowable fractional change. Under the SLTS method, the larger physical time step and the faster speed of convergence can be achieved for the fluid simulations. However, SLTS method is not suitable for aeroacoustic simulations even when the convergence criteria are satisfied because of the Newton linearization error of the term $\partial U_{p} {/}\partial \tau$. Hence, adaptively switched time stepping scheme (ASTS)^24,25^ was applied to maintain the accuracy in the aeroacoustic simulation at the same time reducing the computational cost.

In the calculation of $R^{k}$ on the right-hand side of Eq. (10), the terms involving *F*_*i*_ in Eq. (3) can be divided into an inviscid term *F*_*inviscid*_, and a viscous term *F*_*viscous*_, as shown below:

13 $$ F_{inviscid} = \left( \begin{gathered} \rho u_{i} \hfill \\ \rho u_{i} u_{1} + p\delta_{i1} \hfill \\ \rho u_{i} u_{2} + p\delta_{i2} \hfill \\ \rho u_{i} u_{3} + p\delta_{i3} \hfill \\ (\rho e + p)u_{i} \hfill \\ \end{gathered} \right), $$

14 $$ F_{viscous} = - \left( \begin{gathered} 0 \hfill \\ \mu A_{i1} \hfill \\ \mu A_{i2} \hfill \\ \mu A_{i3} \hfill \\ \mu A_{ij} u_{j} + \lambda \frac{\partial T}{{\partial x_{i} }} \hfill \\ \end{gathered} \right), $$

When employed the Roe scheme in Eq. (14), *F*_*inciscid*_ term will be discretized into

15 $$ F_{inviscid,i + 1/2} = \frac{1}{2}[F_{R} (U) + F_{L} (U)] + F_{d} , $$

where *F*_*d*_ is the Roe dissipation term, which is composed of jumps of properties of work fluids. For the reconstruction of *F*_*R*_ and *F*_*L*_, we adopted the fifth-order monotone upstream-centered scheme for conservation laws (MUSCL)^26^ without a limiter function to prevent turbulent fluctuations from attenuating. In addition to the inviscid term, the derivative terms in *A*_*ij*_ in the viscous term of Eq. (3) are calculated using the second-order central difference. The detail of the current framework can be found in previous study^27,28^.

### Computational conditions

**Figure 3:**
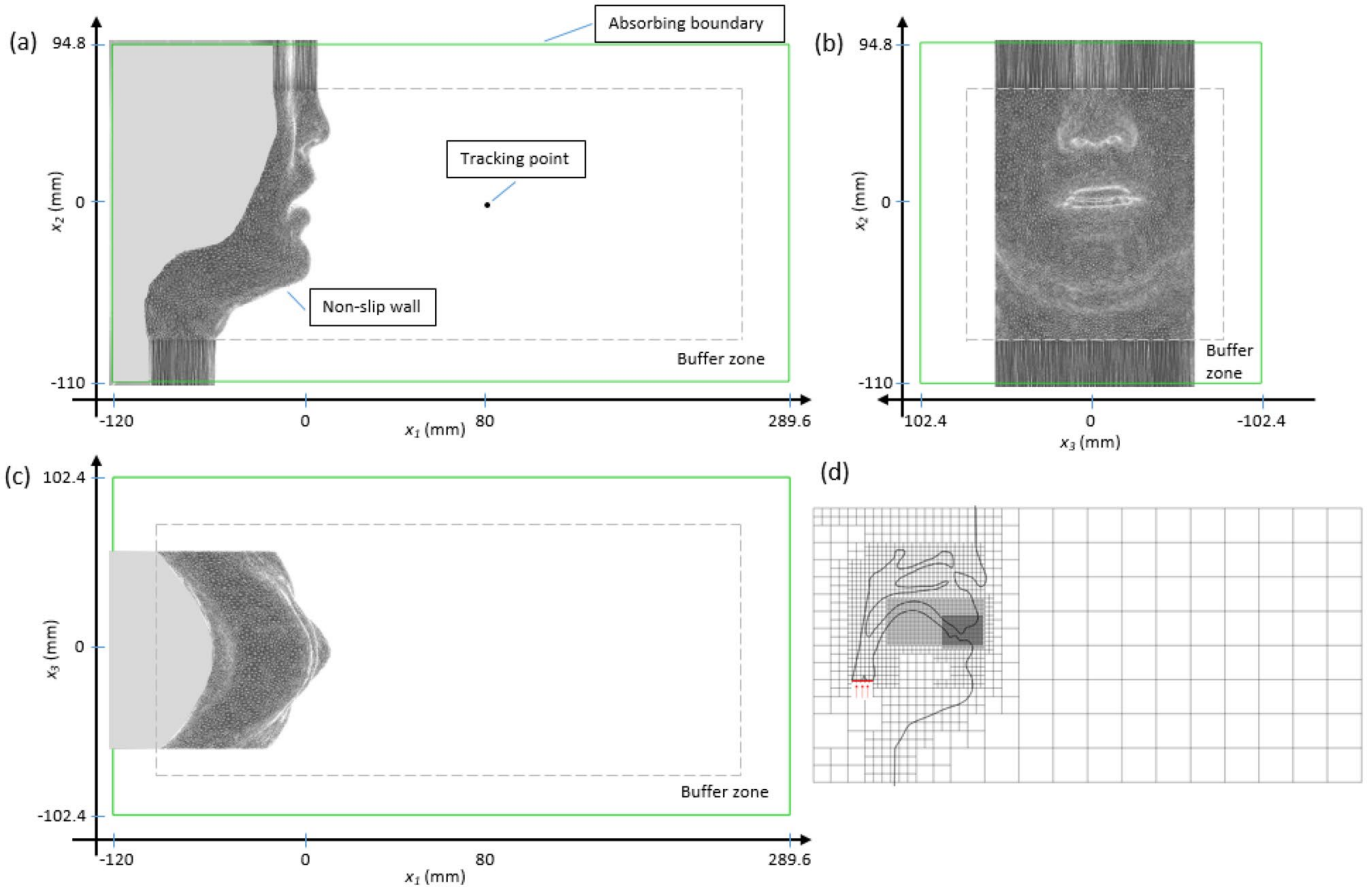
(**a**)–(**c**) The three-view diagram of overall computational domain. (**d**) grid distribution (every 16th gridline is shown for clarity).

The three-view diagram of the overall computational domain is shown in Fig. 3a–c. The *x*_1_ is defined as the anterior–posterior direction; the *x*_2_ is defined as the inferior–superior direction; the *x*_3_ is defined as the transverse direction. The time step was set as 2 × 10^−6^ s and the minimum grid size was chosen to 0.05 mm. The grid convergence was tested and the computational accuracy is enough to capture the acoustic characteristics of /s/, as described in the supplementary information. The total grid number was approximately 1.82 × 10^8^. The grid distribution on the mid-sagittal plane is shown in Fig. 3d. The immersed boundary method was adopted for the geometry of the realistic human vocal tract and the tongue and velum movement during the articulatory process. To reduce the grid generation time for the geometry and to provide better load balancing and higher performance on parallelization, the hierarchical structure grid^29^ was applied in the simulations. Using the hierarchical structure grid system can make the working time required to build the computational grids shorter and simultaneously provide better load balancing and higher performance for parallel computations.

Although it is different from the physiologically changing flow rate in real phonation process, the constant flow rate was set to focus on the effect of the movement of the speech organ. The uniform inlet velocity was set to 1.5 m/s as the red line shown in Fig. 3d, which resulted in a physiological flowrate of 450 cm^3^/s. The sibilant /s/ sound will be generated by the geometry of the vocal tract automatically, therefore, there is no voice source set in this simulation. To keep the flow in the computational domain from being polluted by reflecting pressure waves at the outer computational boundary, an absorbing boundary condition was used as the outlet condition. The utilized absorbing boundary condition is based on Freund^30^ and extended by Li^28^ which is adjusted for the low Mach number simulation. Fast Fourier transform (FFT) using the Hann window was applied to the pressure waveforms sampled at 80 mm from the lip outlet to analyze the far-field sound spectrogram. The FFT sampling frequency was 50 kHz with 256 points. The overlap ratio was 0.9 for the spectrogram calculation. The sound pressure level (SPL) was calculated based on the reference pressure *P*_*ref*_ = 20 × 10^−6^ Pa.

## Results and discussion

**Figure 4:**
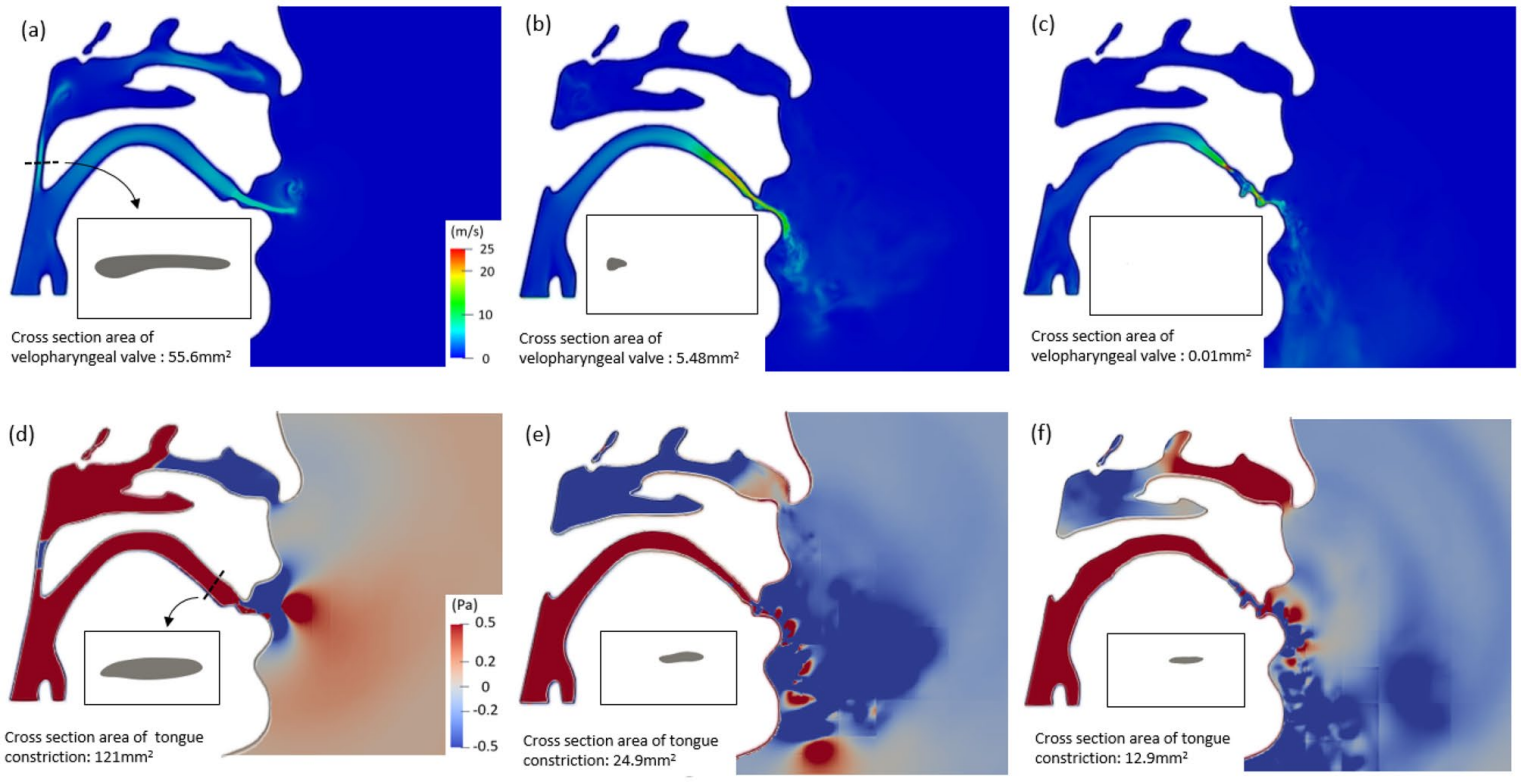
(**a**)–(**c**) Instantaneous flow field and (**d**)–(**f**) pressure fluctuation along the sagittal plane at (**a**, **d**) *t* = 0.01 s, (**b**, **e**) *t* = 0.05 s and (**c**, **f**) *t* = 0.10 s.

The instantaneous flow field and the pressure fluctuation along the sagittal plane from /u/ to /s/ are shown in Fig. 4 for the original motion of the CT scans with the velum speed of 0.208 m/s. At *t* = 0.01 s (Fig. 4a,d), the tongue was at the lower position so that the size of the tongue constriction was not small enough to produce the sound of /s/. In addition, the velopharyngeal valve was opened which let the airflow go to both nasal and oral cavities. Specifically, there was 35% airflow go through the velopharyngeal valve and 65% airflow goes to the oral cavity. Therefore, the velocity of the mainstream at the oral cavity was only around 10 m/s and there was no sound propagation from both nasal and oral cavities. At *t* = 0.05 s (Fig. 4b,e), since the velopharyngeal valve kept closing the channel to the nasal cavity, the higher flow resistance decreased the flow rate from 35 to 13% in the nasal cavity and increased the flow rate from 65 to 87% in the oral cavity. Moreover, the elevated tongue position caused the reduction of the cross-sectional area of the oral cavity so that the velocity of the mainstream in the oral cavity was accelerated to around 20 m/s and the periodic pressure wave was observed near the lips. Besides, because of the narrow velopharyngeal valve, the velocity became turbulent at the nasal cavity which resulted in the pressure wave at the exit of the nose in Fig. 4e. At *t* = 0.1 s (Fig. 4c,f), the tip of the tongue moved posterior direction and elevated towards the hard palate. The velocity of the mainstream in the oral cavity reached around 25 m/s and the amplitude of the sound was increased at the far-field. At this time, since the velopharyngeal valve was completely closed, there was no nasal sound emission.

**Figure 5:**
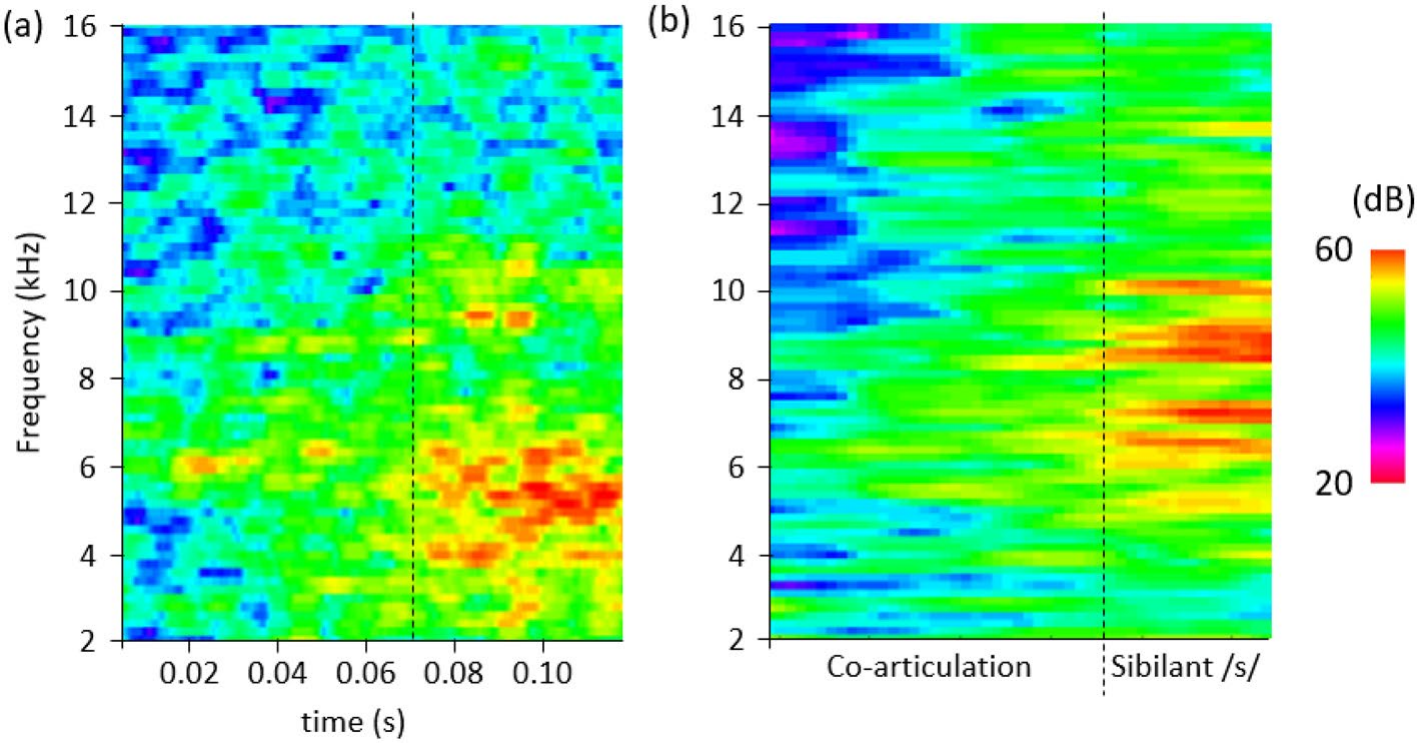
The spectrogram during co-articulation and sibilant /s/ sound (**a**) by the current study (**b**) measurement for the subject of the CT scan^25^.

The spectrogram of the propagating sound collected at 80 mm from the lip outlet, is shown in Fig. 5a. For *t* = 0.01 s to *t* = 0.03 s, since the cross-sectional area of the oral tract was deceased with the tongue elevation and velopharynx closure, the amplitude peak at 6 kHz gradually appeared after *t* = 0.02 s. The amplitude of the broadband noise around 5–9 kHz was observed with relatively small amplitudes since the velopharyngeal valve was still open and some of the airflows kept going through the nasal cavity. For *t* = 0.03 s to *t* = 0.07 s, because of the closing velopharyngeal valve, the broadband noise was amplified in the extended frequency range of 3–11 kHz. After *t* = 0.07 s, when the size of the velopharyngeal valve became smaller than that of the tongue constriction, the airflow tended to go to the oral cavity rather than the nasal cavity. Since the tongue constriction at this moment was narrow enough for the production of the sibilant sound, the typical sibilant sound was generated with the characteristics peak around 4–6 k Hz, and the broadband noise was observed around 4–12 k Hz. Although the frequency range of the /s/ sound is different, the spectrogram during the co-articulation is similar to that shown in Fig. 5b which was pronounced by the subject of CT scan^31^. Furthermore, the relation between the size of the velopharyngeal valve and the sound amplitude observed at the far-field was consistent with the previous studies^11,14,15^.

**Figure 6:**
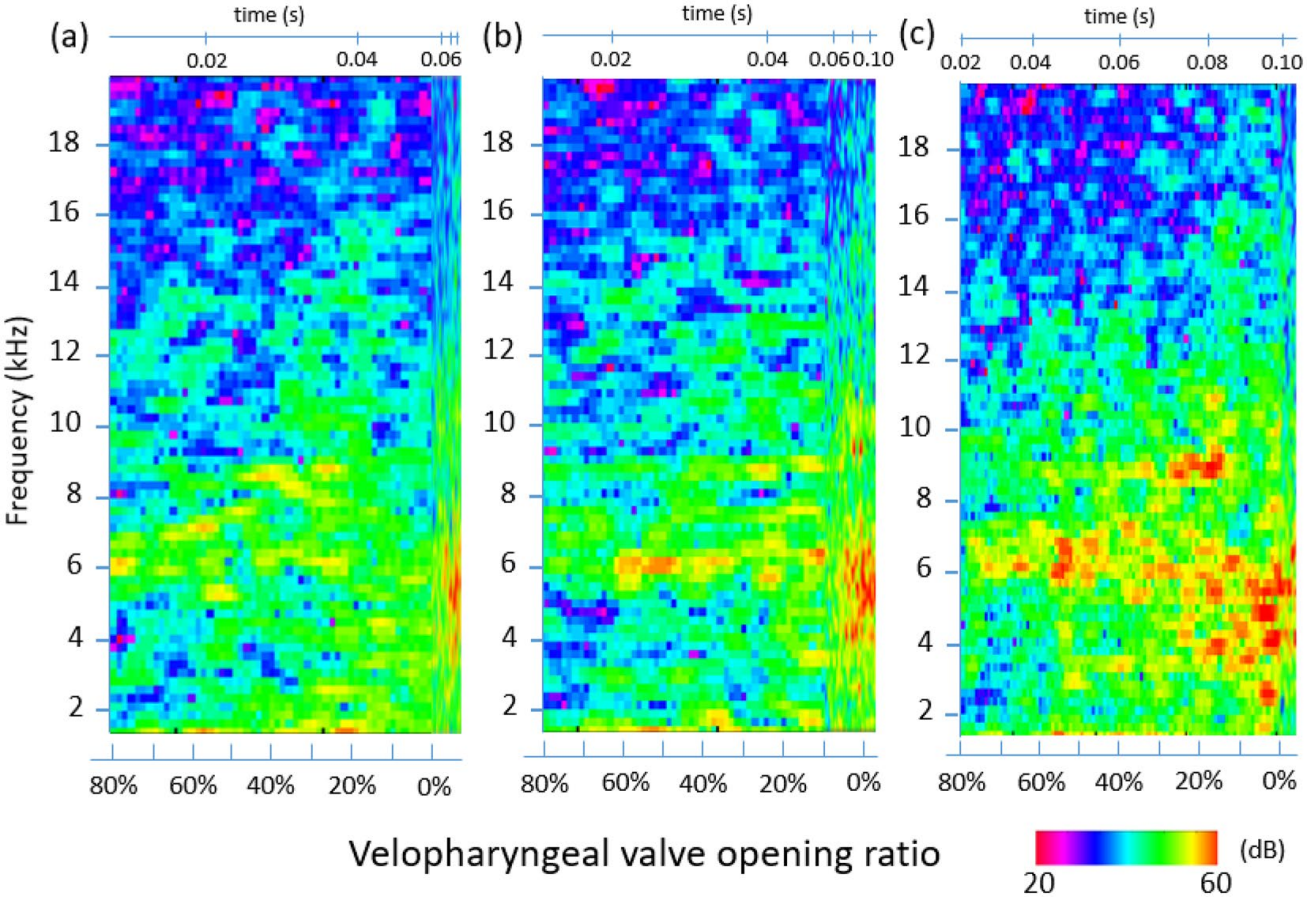
The far-field spectrogram with the closing speed of (**a**) 0.220 m/s (faster closing case), (**b**) 0.208 m/s (base simulation) and (**c**) 0.152 m/s (slower closing case) base on the velopharyngeal valve opening ratio.

To investigate the effect of the closing speed of the velopharyngeal valve, simulations with closing speeds of 0.220 m/s, 0.208 m/s and 0.152 m/s were conducted with the cross-sectional area ratio shown in Fig. 2b. To clarify the relationship between the sound amplitude and velum closing speed, the spectrograms of the different closing speeds are shown in Fig. 6 base on the velopharyngeal valve opening. The horizontal axis is plotted with the velum opening ratio, and the complete closure (0% opening) was around *t* = 0.05 s for the case with 0.220 m/s, *t* = 0.09 s for the original case, and *t* = 0.10 s for the case with 0.152 m/s. As seen in the figure, at 80% valve opening ratio, peaks were mainly observed around 6 kHz in all cases. While the valve opening ratio reached 20%, the frequency range of the broadband noise was increased to 3–10 kHz in all cases. Although the amplitudes of the three cases were different, the tendency of the broadband noise indicated that the sound is strongly related to the opening of the velopharyngeal valve. Besides, the amplitudes of 0.152 m/s in the frequency range 3–7 kHz were larger than those of 0.208 m/s and 0.220 m/s.

**Figure 7:**
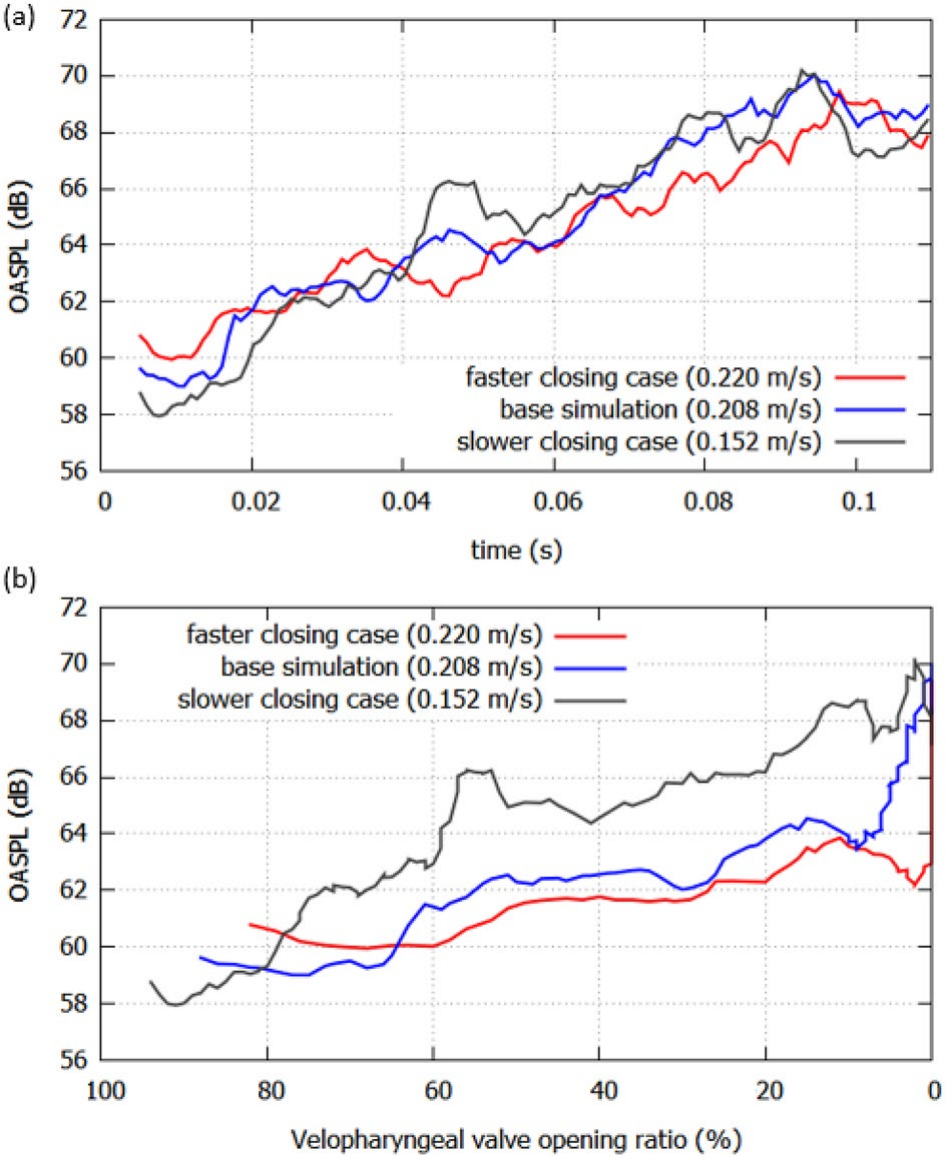
(**a**) The OASPLs with the different closing speed. (**b**) The OASPLs based on the velopharyngeal valve opening ratio.

The overall sound pressure level (OASPL) values from 1.5 to 20 kHz during the articulatory process are shown in Fig. 7a. The OASPL of the 0.220 m/s was larger than the other cases around *t* = 0.01 s, because of the higher flow rate through the oral cavity caused by the higher flow resistance in the nasal cavity. However, the OASPL of 0.152 m/s became larger than that of 0.220 m/s after *t* = 0.04 s. The amplitude of 0.220 m/s at this moment became the lowest since the velopharyngeal valve was totally closed. On the contrary, because the channel to the nasal cavity was still 50% opened for 0.152 m/s, the audible nasal emission made the OASPL higher at *t* = 0.05 s. To identify the effect of the velum closure speed, the OASPL based on the velopharyngeal valve opening ratio was shown in Fig. 7b. As shown in the figure, the amplitude was amplified for 0.152 m/s in the opening ratio from 75 to 5%. The mean OASPL of the 0.220 m/s, 0.208 m/s, and 0.152 m/s are 61.8 dB, 62.1 dB, and 64.2 dB, respectively, during the articulatory process (*t* = 0–0.1 s). At the velum opening of 55%, the OASPL of 0.152 m/s was 5 dB louder than that of 0.220 m/s because the area ratio of velopharyngeal to oral channel was larger in slower closing case.

**Figure 8:**
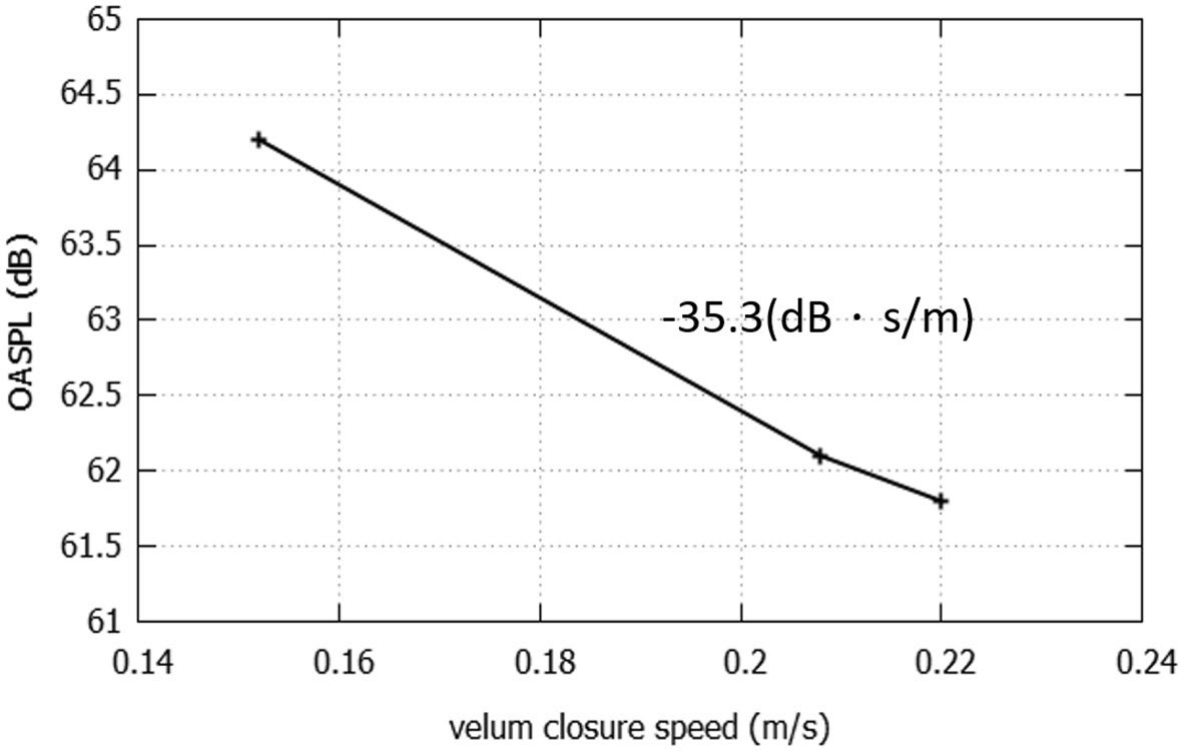
The relation between OASPLs during the phonation process and the velum closure speed.

Moreover, the relation between the mean OASPLs and the velum closure speed was plotted in Fig. 8. The relation was in the inverse proportion with the slope equaled to − 35.3 dB·s/m. This indicated that the sound generated before the /s/ will be enlarged while the slower closing speed of the velopharyngeal valve. This amplified sound might be related to area ratio of velopharyngeal to oral constriction mentioned by Sundström^11^. Consequently, the slower velum closure speed will result in the larger sound amplitude during the co-articulation and might disturb the acoustic characteristics of pronounce sounds. Although we performed the simulation only for this subject, further simulation with additional patients with hypernasality may help the diagnosis of the velopharyngeal insufficiency.

## Conclusion

To investigate the effects of the velopharyngeal closure speed and the tongue constriction formation on the sibilant sound production, a numerical simulation of the articulatory process after the vowel /u/ to the sibilant sound /s/ was conducted. The broadband noise generation during the co-articulation process was observed and consistent with the measurement result. To clarify the effect of velopharyngeal closure speed, the simulations with different velum closing speed were conducted. The spectrograms based on the velopharyngeal valve opening showed that the frequency range of the broadband noise of /s/ was extended from 6 to 3–7 kHz during the co-articulation process in all cases, and this indicated that the sound amplitude is strongly related to the velopharyngeal opening. In addition, the sound amplitude increased in the case with the slower velum closure during the co-articulation process, indicating the possible emission of indistinguishable sound. These findings can provide the underlying insights necessary to the oral surgical treatment for generating the sibilant sound. Moreover, the present framework will enable us to predict the effects of the vocal tract shapes for more subjects on the production of sibilant fricatives with shorter time to build the database which benefits patients prior to prosthetic surgery.

## Supplementary Information

Supplementary Information.

## Data Availability

The datasets generated or analysed during the current study are available from the corresponding author on reasonable request.
