# Supplementary material for: Numerical investigation of effects of tongue articulation and velopharyngeal closure on the production of sibilant [s]
Source: Sci Rep. 2022 Sep 13;12:15361. doi: 10.1038/s41598-022-18784-7 (PMC9470661; doi:10.1038/s41598-022-18784-7)
Supplement: Supplementary file 1 — Supplementary Information. [file 41598_2022_18784_MOESM1_ESM.docx]

Supplementary Information: Grid Convergence

Numerical investigation of effects of tongue articulation and velopharyngeal closure on the production of sibilant [s]

HsuehJui Lu, Tsukasa Yoshinaga, ChungGang Li*, Kazunori Nozaki, Akiyoshi Iida and Makoto Tsubokura

The convergence test with the different grid configuration had been done with the sibilant /s/. Two cases with the same minimum grid size of 0.05 mm were conducted. The total grid number of case 1 was approximately 47 million. On the other hand, due to the finer resolution area was extended from the tongue constriction to the front part of the vocal tract, the total grid number of case 2 was 96 million which was twice of case 1. The SPL simulated at 10 cm from the mouth with two grid cases and the experimental^1^ results are shown in Figure A1. The amplitudes of two cases were similar in the frequency range above 5 kHz. However, in the frequency range below 5 kHz, case 1 overestimated the experimental values, and the first characteristic peak of /s/ was shifted to lower frequency. In contrast, the amplitudes of case 2 started increasing at 2 kHz and reached the first peak at 5 kHz in the similar way as the experimental measurement. This result suggests that keeping fine resolution at the front part of the vocal tract is important to capture the characteristics of /s/, and therefore, the grid configuration of case 2 had been chosen in this study.


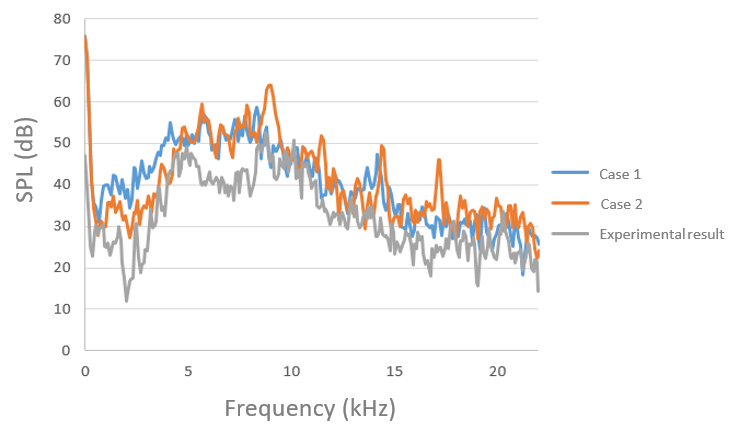


Figure A1. Sound pressure level (SPL) spectrum of the sibilant /s/.

^1^ Yoshinaga, T., Nozaki, K., & Wada, S. (2019). A simplified vocal tract model for articulation of [s]: The effect of tongue tip elevation on [s]. PloS one, 14(10), e0223382.
